# HPV DNA integration site as proof of the origin of ovarian metastasis from endocervical adenocarcinoma: three case reports

**DOI:** 10.1186/s12885-019-5582-8

**Published:** 2019-04-23

**Authors:** Alexandra Arfi, Delphine Hequet, Guillaume Bataillon, Carine Tran-Perennou, Fereshteh Farkhondeh, Xavier Sastre-Garau, Virginie Fourchotte, Roman Rouzier, Enora Laas, Nicolas Pouget, Anne Vincent-Salomon, Emmanuelle Jeannot

**Affiliations:** 1 Department of Surgery, Institut Curie, 92210 St Cloud, France; 2 Institut Curie, Inserm U900 – Bioinformatics, biostatistics, epidemiology and computational systems. Cancer biology, 35, rue Dailly, 92210 Saint-Cloud, France; 3 Department of Pathology, Institut Curie, 75005 Paris, France; 4 Department of Pathology, Institut de Cancérologie de Lorraine, 54519 Vandoeuvre-Lès-Nancy, France

**Keywords:** Cervical neoplasia, Ovarian metastasis, p16, HPV, Integration

## Abstract

**Background:**

Most endocervical adenocarcinomas are human papillomavirus (HPV)-related cancers associated with p16 immunostaining. Ovarian metastasis from cervical cancer is a rare phenomenon, the mechanism of dissemination remains unclear. The diagnosis of metastasis may be difficult to establish when the ovarian neoplasm presents features consistent with primary tumor. Immunohistochemical expression of p16 in ovarian tumors can guide the diagnosis of metastasis from HPV-related cervical cancer, but p16 positivity is nonspecific. Identical HPV genotype in the paired endocervical and ovarian tumors is a better marker for cervical origin, which may also be confirmed by identical HPV integration site.

**Case presentation:**

Two women presented with HPV18 cervical adenocarcinoma. No signs of disease were visible on MRI after treatment. After several years of follow-up, mucinous ovarian tumors were discovered in both patients. Molecular analyses showed that the ovarian lesions were HPV18-positive; indicating a primary cervical origin. A third woman was diagnosed with grade 1 ovarian endometrioid carcinoma with no peritoneal carcinomatosis. Final histological examination and HPV genotyping revealed HPV18-related in situ endometrioid adenocarcinoma in the endocervix and HPV18-related invasive endometrioid adenocarcinoma in the endometrium and both ovaries. Additional molecular analyses performed in two patients identified the same HPV integration sites in both the ovarian and cervical tumors, confirming that the ovarian mass was a metastasis from the cervical adenocarcinoma.

**Conclusion:**

We report three new cases of ovarian neoplasia in which the diagnosis of metastasis from cervical cancer was supported by the same HPV genotype and the same integration site in the paired cervical and ovarian tumors. To our knowledge, this is the first report of molecular evidence of the cervical origin of an ovarian metastasis. HPV screening should be performed in ovarian tumors for all patients with history of cervical neoplasia.

## Background

Endocervical adenocarcinomas are high-risk human papillomavirus (HPV)-related cancers. HPV16 or HPV18 are the genotypes most frequently observed [1]. HPV-related endocervical adenocarcinomas are also associated with p16 expression, and loss of hormone receptor expression is usually observed [2]. Typically, cervical adenocarcinomas primarily spread to adjacent pelvic structures and to pelvic lymph nodes. Metastasis to the ovaries is an infrequent event, but a known risk. Diagnosis of metastatic disease is difficult, as the ovarian tumor presents features consistent with primary tumor, such as mucinous or endometrioid histology [3].

Only a few cases of HPV-related ovarian metastasis from endocervical adenocarcinoma have been reported in the literature over the last decade [3, 4]. In these reports, the evidence of metastasis was supported by the presence of identical HPV genotypes in the paired endocervical and ovarian tumors. The mechanism of metastasis still remains unclear, and additional proof of the cervical origin is needed. In this context, HPV integration site is a specific marker of cervical origin, as HPV genome integration into tumor DNA constitutes a different event from cervical tumor to another [5, 6].

In the present report, we describe our experience of three cases and provide proof of the cervical origin of the metastasis, based on identification of the same integration site of the HPV genome in the paired endocervical and ovarian tumor DNAs. To our knowledge, this is the first time that tumor spread from endocervix to ovary has been demonstrated by molecular evidence.

## Case presentation

### Case 1

A 55-year-old nulliparous postmenopausal woman with no medical history was managed for FIGO (International Federation of Gynecology and Obstetrics classification) stage IIIB cervical cancer. Cervical biopsies showed HPV18-related moderately differentiated invasive adenocarcinoma. Magnetic resonance imaging (MRI) revealed a 4 cm anterior mass extending to the uterine isthmus, uterine corpus, left parametrium and superior third of the vagina. No other lesion was visualized on abdominal computed tomography (CT) and positron emission tomography (PET-CT). Concomitant external beam pelvic radiation (45 Gray (Gy) in 1.8 Gy daily fractions) and six cycles of chemotherapy (weekly cisplatin 40 mg/m^2^) were administered. The patient was reevaluated by MRI at the end of treatment, showing less than 50% size response with persistent parametrial involvement. Adjuvant brachytherapy (25 Gy) and external beam pelvic radiation (8 Gy in 3 daily fractions) was therefore decided. Two months later, the lesion had completely resolved on MRI. After 3 years of follow-up, MRI revealed a pelvic mass with no increased uptake on PET-CT. Bilateral salpingo-oophorectomy was performed based on a diagnosis of right ovarian mass without peritoneal carcinomatosis or other distant disease. Histological examination concluded on invasive mucinous adenocarcinoma.

**Fig. 1:**
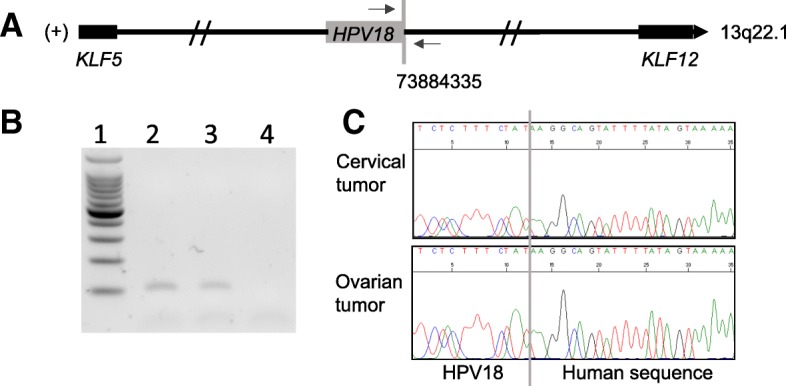
Identification of HPV integration site in 13q22.1 in both cervical and ovarian tumors, case 1. **a** The junction between HPV18 (grey box) and human genome is indicated by a vertical grey line. The coordinate of the human genome breakpoint (73884335) in cervical tumor was determined according to the forward strand (+) of hg19 reference. Horizontal arrows indicate the localization of the primers, which were designed to specifically amplify the human/viral junction sequence. The closest genes (KLF5, KLF12) to the HPV integration site are shown as black boxes. **b** Analysis of the PCR products on agarose gel. Lane 1 represents molecular weight, lanes 2 and 3 indicate cervical and ovarian tumor DNA, respectively. Lane 4 is DNA from another patient (negative control). **c** Sanger sequencing chromatogram obtained from cervical tumor DNA (upper part) and ovarian tumor DNA (lower part). Vertical grey line indicates the junction between HPV18 (left part) and the human genome (right part)

To determine the origin of the ovarian lesion and in view of the synchronous HPV18-positive cervical carcinoma, molecular analyses were performed, showing that the ovarian tumor was HPV18-positive, strongly suggesting a primary cervical origin. As previous analyses of the cervical tumor identified the HPV integration site in chromosome 13, the ovarian tumor was screened to determine whether the ovarian metastasis presented the same HPV integration site. The same HPV integration site at locus 13q22.1 was demonstrated in ovarian tumor DNA, clearly confirming that the ovarian mass was a metastasis from the cervical adenocarcinoma (Fig. 1). Six cycles of chemotherapy (weekly paclitaxel and carboplatin) were therefore administered. Eighteen months later, the patient presented recurrence in the form of peritoneal carcinomatosis and a rectal nodule confirmed histologically at laparoscopy. Treatment of the recurrence consisted of six cycles of carboplatin and paclitaxel, which was early stopped because of neuropathy toxicity. After a therapeutic break requested by the patient, she was included in a phase 1 study for advanced gynecological cancers (clinicaltrials.gov identifier: NCT 02978755). The patient is still on treatment.

### Case 2

A 45-year-old smoking primiparous woman with no medical history presented with ascites and a left ovarian mass. Histological examination of the mass revealed grade 1 ovarian endometrioid carcinoma. Staging surgery with total hysterectomy, bilateral salpingo-oophorectomy, omentectomy, and pelvic and para-aortic lymphadenectomies were therefore performed. No peritoneal carcinomatosis was observed.

**Fig. 2:**
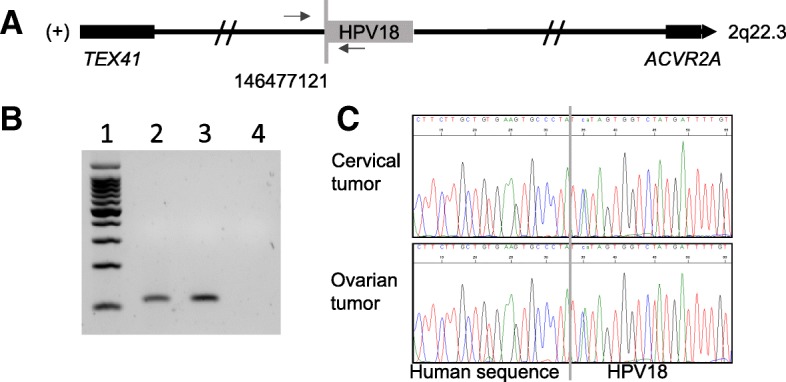
Identification of the HPV integration site in 2q22.3 in both cervical and ovarian tumors, case 2. **a** The junction between HPV18 (grey box) and the human genome is indicated by a vertical grey line. The coordinate of the human genome breakpoint (146477121) in the cervical tumor was determined according to the forward strand (+) of the hg19 reference. Horizontal arrows indicate the sites of the primers designed to specifically amplify the human/viral junction sequence. The genes closest to the HPV integration site are identified at 643 kb (TEX41) and 2 Mb (ACVR2) and shown as black boxes. **b** Analysis of the PCR products on agarose gel. Lane 1 represents molecular weight, lanes 2 and 3 indicate cervical and ovarian tumor DNA, respectively. Lane 4 is DNA from another patient (negative control). **c** Sanger sequencing chromatogram obtained from cervical tumor DNA (upper part) and ovarian tumor DNA (lower part). The vertical grey line indicates the junction between HPV18 (left part) and the human genome (right part)

HPV18-positive in situ endometrioid adenocarcinoma was found in the endocervix and HPV18-positive invasive endometrioid adenocarcinoma was found in the endometrium and both ovaries on final histological examination. The same HPV integration site in locus 2q22.3 was demonstrated in the ovarian tumor DNA, clearly confirming that the ovarian mass was a metastasis from the cervical adenocarcinoma (Fig. 2). The patient was then treated with radiotherapy and brachytherapy.

### Case 3

A 30-year-old nulliparous woman with no medical history, regularly screened by PAP-smear test presented with postcoital metrorrhagia. In situ carcinoma was diagnosed on cervical biopsies. MRI and abdominal CT imaging were normal. A large loop cervical excision and endometrial curettage were performed and confirmed the presence of a well differentiated HPV18-related in situ adenocarcinoma and high-grade cervical intraepithelial neoplasia (CIN III) with negative surgical margins. No obvious infiltration was observed on the examined slides. Nine years later, after several failures of in vitro fertilization, the patient experienced abdominal pain. Ultrasound imaging revealed a 9.5 cm complex left adnexal mass. Examination of the laparoscopic left salpingo-oophorectomy revealed mucinous cystadenoma of the intestinal type with borderline character traits and extensive foci of intraepithelial carcinoma. Seventeen months later, after being lost to follow-up, a 10 cm right ovarian mass was discovered. Total hysterectomy, right salpingo-oophorectomy, appendectomy, omentectomy were performed, revealing a 2.5 cm HPV18-related cervical cancer extending to the uterine isthmus with mucinous proliferation of intestinal type involving the cervix and right ovary. The HPV insertion site could not be determined due to insufficient tumor tissue. One month later, the patient was referred to our oncologic surgery department, where complementary laparoscopic pelvic and para-aortic lymphadenectomies were performed. Carcinomatous metastasis and intestinal involvement were discovered during laparoscopy. The patient was treated with FOLFOX chemotherapy for 3 months and then underwent complete cytoreductive surgery with peritonectomy, multiple bowel resections and intraperitoneal hyperthermic chemotherapy due to a partial response to chemotherapy. Examination of the resected tissues showed carcinomatous cells partly modified by chemotherapy and a high mitotic index (Ki 67: 70%). The patient is currently on follow-up.

### HPV genotyping and integration site

Total DNA, isolated from formalin-fixed tissue blocks, was used for HPV typing. Real-time PCR using Sybr®Green PCR Master mix at a final concentration of 1X, specific primers for HPV16, HPV18 and HPV33 [2] at a final concentration of 660 nM each and 10 ng of tumor DNA was performed on a 7900HT Fast Real-Time PCR System in a final volume of 26 μL (Applied Biosystems, Foster City, CA). PCR reaction were run under the following program: 95 °C 15 min, 40 cycles of (95 °C 1 min, 55 °C 1 min, 72 °C 1 min), followed by a dissociation stage (95 °C 15 s, 60 °C 1 min, 95 °C 15 s).

For the identification of HPV integrations sites in patient 1 and patient 2, the DIPS-PCR method was performed on total DNA isolated from cryopreserved tumor tissue [7]. Briefly, after overnight ligation to an adapter, digested DNAs were submitted to two runs of PCR. For the analysis, 10 HPV18 primers matching the frequent viral breakpoint areas (E1, E2, L1, and L2 genes) were used. PCR products were run on an agarose gel and selected amplicons were analyzed by Sanger sequencing. Genomic chromosome-viral breakpoints were identified by alignment of the sequence with the HPV18 genome using the nucleotide BLAST tool from NCBI (https://blast.ncbi.nlm.nih.gov/Blast.cgi) and human genome (hg19 reference) using the blat tool (https://genome.ucsc.edu).

Presence of HPV of the same integration site in both cervical and ovarian tumors were confirmed by PCR using specific primers followed by Sanger sequencing, as shown by Figs. 1 and 2, parts B and C.

### P16 staining

Immunohistochemistry was performed for p16 on formalin-fixed, paraffin-embedded tissue sections using the prediluted E6H4 antibody (Ventana Medical Systems, Inc.) on a Leica Bond automated instrument (Leica Biosystems, Inc.) with standard antigen retrieval and incubation times. Both nuclear and cytoplasmic staining was required for a cell to be considered “positive”.

## Discussion and conclusions

We describe, for the first time, the molecular signature of an HPV18-positive ovarian metastasis from cervical carcinoma based on identification of the same HPV DNA integration site in both cervical and ovarian cancer cells.

Ovarian metastasis from cervical adenocarcinoma is a rare phenomenon, as cervical adenocarcinoma usually gives rise to metastases in contiguous structures and pelvic lymph nodes. The differential diagnosis with primary mucinous ovarian carcinoma is challenging [3]. Similar HPV subtypes in both ovarian and cervical tumors strongly support the diagnosis of ovarian metastasis from primary cervical cancer [8]. According to Shimada et al., the incidence of ovarian metastasis from cervical adenocarcinoma is 5.31% and the presence of ovarian metastasis does not correlate with lymph node involvement or parametrial invasion [9]. Thirty-four cases of HPV-related ovarian metastasis with cervical adenocarcinoma have been described in the literature [3, 4, 10, 11]: 25 patients (73.5%) were diagnosed with an invasive cervical tumor and 9 patients (26.5%) were diagnosed with an in situ cervical tumor (Table 1). Twenty-nine (85.3%) of these patients were diagnosed with borderline ovarian metastasis and 5 (14.7%) with invasive ovarian metastasis (Table 1).

**Table 1:** Characteristics of previously published cases and the cases reported here

Studies	Number	Cervical tumor		Ovarian tumor		HPV type			
	of cases	AIS, n	Invasive, n	Borderline, n	Invasive, n	16, n	18, n	45, n	Unknown, n
Ronnett et al. [3]	29	6	23	26	3	16	7	3	3
Chang et al. [11]	2	2	0	1	1	2	0	0	0
Turashvili et al. [10]	2	1	1	2	0	1	0	0	1
Ashton et al. [4]	1	1	0	1	0	0	1	0	0
Present study	3	2	1	1	2	0	3	0	0

AIS: in situ adenocarcinoma; n: number of cases

**Fig. 3:**
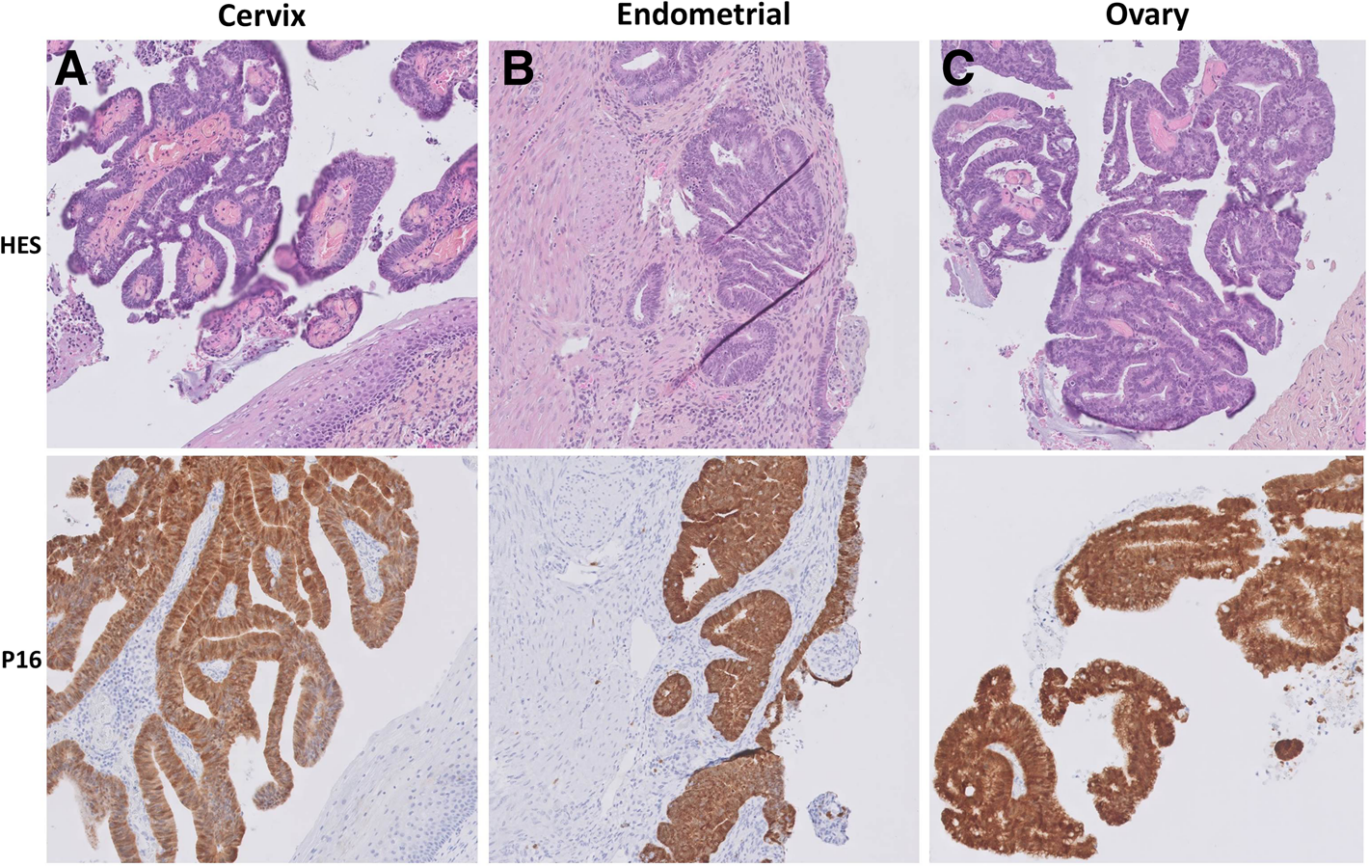
Case 2 In situ endometrioid endocervical adenocarcinoma with focal uterine corpus involvement and synchronous ovarian metastasis. **a** Tumor in lower endocervix is composed of endometrioid endocervical adenocarcinoma with no stromal invasion. **b** Invasive adenocarcinoma implant in uterine endomyometrium simulates primary endometrial endometrioid carcinoma. **c** Ovarian metastasis demonstrates an intracystic papillary and cribriform growth pattern simulating a primary ovarian tumor. All tumor sites displayed diffuse and strong p16 expression

HPV-related cervical adenocarcinomas typically express P16 [12], a surrogate marker of high-risk HPV infection [13]. Diffuse moderate-to-strong P16 expression has been shown to be a sensitive (100%) and specific (97%) marker for identification of ovarian metastasis from HPV-related cervical adenocarcinoma [8]. In the cases presented here, standard hematoxylin-eosin-safran (HES) staining and p16 immunohistochemical staining were performed on cervical, endometrial and ovarian biopsies. All tumor sites displayed diffuse and strong p16 expression (Fig. 3). However, Yoon et al. have recently shown that p16 is also expressed in primary ovarian cancer and borderline ovarian lesions, with diffuse, moderate-to-strong p16 immunoreactivity [14]. In the light of these new data, p16 expression appears to be a strong but not sufficiently specific marker of ovarian metastasis from HPV-related cervical cancer.

These case reports demonstrate the very specific molecular signature of the HPV integration site and the filiation between the cervical carcinoma and the ovarian secondaries. Three hypotheses have been proposed to explain dissemination of cervical adenocarcinoma to the ovary [3, 11, 15]: lymphovascular/hematogenous spread of cervical cancer cells, retrograde uterine/transtubal spread of cervical cancer cells, and retrograde uterine/transtubal spread of HPV infection. However, these last two hypotheses remain controversial. Shimada et al. showed that 7 out of 9 patients presented both lymphatic and hematogenous metastases on histological examination [9]. These results suggest that the pathway of invasion and migration of the ovaries by cervical cancer cells may differ according to the histological type. Reichert et al. proposed that cervical cancer cells may ascend from the cervix to the ovaries through the fallopian tubes in premenopausal women at the time of ovulation via a ruptured corpus luteum [15]. However, this mechanism cannot explain ovarian metastases in postmenopausal patients.

As the same genetic alteration was identified in both cervical and ovarian tumors, we can rule out the hypothesis proposed by Reichert et al. based on ascending spread of HPV infection [15], as HPV DNA integration at exactly the same site in the host cell would be extremely unlikely. Identification of the same nucleotide breaks in both tumor and viral DNA in cervical and ovarian tumors confirms that HPV-related tumor cells may spread from the cervix to the ovary.

HPV genotyping, performed with commercial kits or in-house assay, is a fast method which works on tumor DNA from FFPE samples and may be good enough to diagnose metastasis of HPV-related tumor. Identification of the same HPV integration site by DIPS-PCR assay in two different tumors clearly demonstrates the filiation between these tumors and rules out the hypothesis of occurrence of different tumors harboring the same HPV type. However, this method has been validated for HPV16 and HPV18 types only, with tumor DNA from cryopreserved tissue samples [7]. A new NGS approach which has been developed at Institut Curie, allows an extensive characterization of viral sequences [6]. This method can be used for any HPV type and may be useful for the development of clinical applications such as the elaboration of innovative therapies targeting viral sequences [16].

When an ovarian tumor is diagnosed in a patient with a history of cervical cancer, clinicians should eliminate the diagnosis of cervical cancer metastasis. P16 expression is a good marker to suggest ovarian metastasis from HPV-related cervical cancer, but is not sufficiently specific, as it can also be expressed in primary ovarian cancer. Therefore, in a clinical setting of a history of HPV-related cervical cancer, the HPV type and HPV integration site should be assessed in the ovarian tumor and compared to those of the primary cervical cancer whenever possible. The same HPV viral DNA integration site or the same HPV genotype in both tumors confirms the cervical origin of the ovarian metastasis.

## Abbreviations

CIN III: High-grade Cervical Intraepithelial Neoplasia
CT: Computed Tomography
DIPS-PCR: Detection of Integrated Papillomavirus Sequences by ligation-mediated PCR
FIGO: International Federation of Gynecology and Obstetrics classification
Gy: Gray
HES: Hematoxylin-Eosin-Safran
HPV: Human Papillomavirus
MRI: Magnetic imaging resonance
PET-CT: Positron Emission Tomography- Computed Tomography

## References

[1] de Sanjose S, Quint WG, Alemany L, Geraets DT, Klaustermeier JE, Lloveras B, Tous S, Felix A, Bravo LE, Shin HR (2010). Human papillomavirus genotype attribution in invasive cervical cancer: a retrospective cross-sectional worldwide study. Lancet Oncol.

[2] Lombard I, Vincent-Salomon A, Validire P, Zafrani B, de la Rochefordiere A, Clough K, Favre M, Pouillart P, Sastre-Garau X (1998). Human papillomavirus genotype as a major determinant of the course of cervical cancer. J Clin Oncol.

[3] Ronnett BM (2016). Endocervical adenocarcinoma: selected diagnostic challenges. Mod Pathol.

[4] Ashton KA, Scurry J, Tabrizi SN, Garland SM, Otton G, Bowden NA (2015). The problem of late ovarian metastases from primary cervical adenocarcinoma. Gynecol Oncol Rep.

[5] Peter M, Stransky N, Couturier J, Hupe P, Barillot E, de Cremoux P, Cottu P, Radvanyi F, Sastre-Garau X (2010). Frequent genomic structural alterations at HPV insertion sites in cervical carcinoma. J Pathol.

[6] Holmes A, Lameiras S, Jeannot E, Marie Y, Castera L, Sastre-Garau X, Nicolas A (2016). Mechanistic signatures of HPV insertions in cervical carcinomas. NPJ Genom Med.

[7] Luft F, Klaes R, Nees M, Durst M, Heilmann V, Melsheimer P, von Knebel Doeberitz M (2001). Detection of integrated papillomavirus sequences by ligation-mediated PCR (DIPS-PCR) and molecular characterization in cervical cancer cells. Int J Cancer.

[8] Vang R, Gown AM, Farinola M, Barry TS, Wheeler DT, Yemelyanova A, Seidman JD, Judson K, Ronnett BM (2007). p16 expression in primary ovarian mucinous and endometrioid tumors and metastatic adenocarcinomas in the ovary: utility for identification of metastatic HPV-related endocervical adenocarcinomas. Am J Surg Pathol.

[9] Shimada M, Kigawa J, Nishimura R, Yamaguchi S, Kuzuya K, Nakanishi T, Suzuki M, Kita T, Iwasaka T, Terakawa N (2006). Ovarian metastasis in carcinoma of the uterine cervix. Gynecol Oncol.

[10] Turashvili G, Farmer P, Colgan T, Childs T (2015). Human papillomavirus-related ovarian metastasis with Endocervical adenocarcinoma: report of 2 cases and review of literature. J Low Genit Tract Dis.

[11] Chang MC, Nevadunsky NS, Viswanathan AN, Crum CP, Feltmate CM (2010). Endocervical adenocarcinoma in situ with ovarian metastases: a unique variant with potential for long-term survival. Int J Gynecol Pathol.

[12] Zielinski GD, Snijders PJ, Rozendaal L, Daalmeijer NF, Risse EK, Voorhorst FJ, Jiwa NM, van der Linden HC, de Schipper FA, Runsink AP (2003). The presence of high-risk HPV combined with specific p53 and p16INK4a expression patterns points to high-risk HPV as the main causative agent for adenocarcinoma in situ and adenocarcinoma of the cervix. J Pathol.

[13] Ishikawa M, Fujii T, Saito M, Nindl I, Ono A, Kubushiro K, Tsukazaki K, Mukai M, Nozawa S (2006). Overexpression of p16 INK4a as an indicator for human papillomavirus oncogenic activity in cervical squamous neoplasia. Int J Gynecol Cancer.

[14] Yoon N, Yoon G, Park CK, Kim HS (2016). Stromal p16 expression is significantly increased in malignant ovarian neoplasms. Oncotarget.

[15] Reichert RA (2005). Synchronous and metachronous endocervical and ovarian neoplasms: a different interpretation of HPV data. Am J Surg Pathol.

[16] Hellner K, Munger K (2011). Human papillomaviruses as therapeutic targets in human cancer. J Clin Oncol.

